# Student Perceptions of Growth-Facilitating and Growth-Constraining Factors of Practice Placements: A Comparison between Japanese and United Kingdom Occupational Therapy Students

**DOI:** 10.1155/2019/8582470

**Published:** 2019-11-28

**Authors:** Reiko Miyamoto, Dido Green, Peter Bontje, Natsuka Suyama, Nobuo Ohshima, Sally S. A. Fever, Jenny Butler

**Affiliations:** ^1^Faculty of Health Sciences, Tokyo Metropolitan University, Arakawa-ku, Tokyo 116-8551, Japan; ^2^Department of Occupational Therapy, Jönköping University, Jönköping, Sweden; ^3^Faculty of Health & Life Sciences, Oxford Brookes University, Jack Straws Lane Marston, Oxford OX3 0FL, UK; ^4^Occupational Therapy, Division of Clinical Health Sciences, Brunel University London, Uxbridge UB8 3PH, Middlesex, UK; ^5^Graduate School of Health Sciences, Tokyo Metropolitan University, Arakawa-ku, Tokyo 116-8551, Japan

## Abstract

This study compared growth-facilitating and growth-constraining experiences of practice placements as perceived by occupational therapy students from Japan and the United Kingdom (UK). Fifteen students from Japan and 14 from the UK used a nominal group technique (NGT) to rank, individually and in groups, their subjective learning experiences during practice placements. Qualitative analysis and simple tabulation based on ranking of items obtained in the NGT were performed. Five item categories were identified from both Japanese and UK students: self-reflection, the role of supervisor, sense of responsibility, clinical knowledge and skills, and time management. Results showed that all students perceived opportunities for self-reflection and feedback from supervisors as growth facilitating and students' passive attitudes towards requirements of practice placements as growth constraining. Country-specific differences between students were observed in clinical knowledge and skills, sense of responsibility, and time management. Japanese students perceived that preparatory study led to successfully treating clients during placement, and they tended to commit to placement assignments at the expense of time outside. UK students valued working independently with a sense of responsibility but considered time-management problems within their placement hours as growth constraining. These differences can be explained by different social norms and expectations of students from Japan and the UK.

## 1. Introduction

In training occupational therapy students, it is desirable that they acquire a broad knowledge base and relevant clinical experience. In particular, clinical practice placement has been recognized as an integral and critical component of their training [1]. Therefore, for improvement of occupational therapy education, clarification of the factors which facilitate or constrain students' growth towards becoming practitioners is required. In this paper, the factors that students report as prompting their learning are called “growth facilitating” and those reported as hindering their learning are called “growth inhibiting.”

In recent decades, practice placement has changed from the traditional apprenticeship model of being in charge of clients for the duration of practice placement under the guidance of supervisors to one wherein a partnership is created between students and clinical supervisors [2, 3]. Practice placements are no longer seen as opportunities for students to practice occupational therapy skills learned from their supervisors, but more as collaborative learning between students and supervisors to nurture future colleagues who can work in a range of practice areas. This change accompanies the trend towards nontraditional practice placement contexts. For example, nontraditional placements are used in Japan to prepare students for providing services outside institutional medical care and in the community [4] and in the United Kingdom to prepare students for working in novel fields of practice [5]. Internationally, research has suggested that new styles of placement (e.g., role-emerging and project placements) provide students with unique opportunities to take the profession into new practice territories and, in turn, map the future for occupational therapy practice [3].

In addition, a growing number of universities include short-term international exchange studies in their curricula to expose students to diversity and to instill international perspectives in their students. Put differently, practice placements are also changing in response to globalization, with the need to train future occupational therapists who can manage diversity in societies. A grounded theory study of occupational therapy practice placement in an international context found that students' learning included adaptability/flexibility, cultural sensitivity and recognition of the value of interpersonal relationships, and gaining confidence through moving beyond one's comfort zone and through increasing autonomy [6]. However, when considering the impact of international exchange on occupational therapy students, it is also essential to understand the different barriers and facilitating factors to learning for students of different countries.

A few studies have started to explore international aspects of occupational therapy student training. One such study made an international comparison of the effects of problem-based learning (an approach to learning that focuses on dissection and discussion of problems or cases in small groups usually supervised by one or more expert tutor(s) or instructor(s)) [7]. This study reported that students in Scotland tended to use interactive reasoning more than students in the USA (albeit that both groups relied predominantly on procedural reasoning). Another study reported differences regarding feelings of preparedness for practice and further education of newly graduated occupational therapists' from Australia and Aotearoa/New Zealand [8]. Most newly graduated occupational therapists felt somewhat prepared for practice. However, only 17.1% of Australian new graduates and even fewer (8.5%) Aotearoa/New Zealand new graduates felt very well prepared [8]. These authors [8] highlighted the importance of competencies such as evidence-based practice, emphasizing the need to further explore methods to increase feelings of preparedness for practice. The limited research in the area of international comparison between practice placements could in part be due to large variability in duration as well as forms of practice placement [3]. In promoting international exchange, conducting comparisons of experience can give useful information to inform effective learning environments for international students' practice placements. Therefore, if we can compare practice placements internationally, we might be able to shed light on facilitators and constraints to students' learning in different countries. In order to reduce the influence of confounding factors for between-group differences, it is desirable to compare programs that have similar forms and durations of practice placement, provided at a similar academic course stage. This study capitalized on an opportunity that arose when the authors, faculty at a Japanese and a UK university, identified that their respective universities provided clinical practice placements with similar conditions. Accordingly, we designed a study to identify and compare in importance the student-perceived growth-facilitating and growth-constraining factors to learning in practice placements at our respective universities and to understand these similarities and differences qualitatively.

## 2. Materials and Methods

This study involved two university programs, one based in Japan and the other based in the UK. This study was approved by the relevant Ethics Committees at both universities (approval numbers: JPN: 12053, UK: 120672), and full informed written consent was obtained from participants. The aims, procedures, and voluntary participation were explained verbally and in writing to all participants, particularly that their participation (or not) would not affect academic grades. While the identities of participating students were known to the researchers leading the nominal group technique (NGT) groups, no personal data or identifying information was recorded with respect to any response during the NGT session.

### 2.1. Participants

Sixty-five occupational therapy students at the Japanese university A and the UK university B, who had completed all practice placements as stipulated in their respective training curricula, were invited to participate in the study. All researchers were lecturers at universities A and B. The Japanese students (JPN-S) were in their last year of a 4-year university program and had recently completed two 8-week practice placements (total of 16 weeks), each conducted in a different setting. The United Kingdom students (UK-S) were also in their last year, but of a 3-year university program. They had recently experienced a 14-week practice placement, conducted in one setting. The main purpose of these final placements in both countries was to learn basic occupational therapy processes and skills, with students required to assess and provide occupational therapy, under supervision, for more than one client during a set period of time. The two universities also had a similar number of overall practice placement weeks in their programs (Table 1).

### 2.2. Procedures

**Figure 1:**
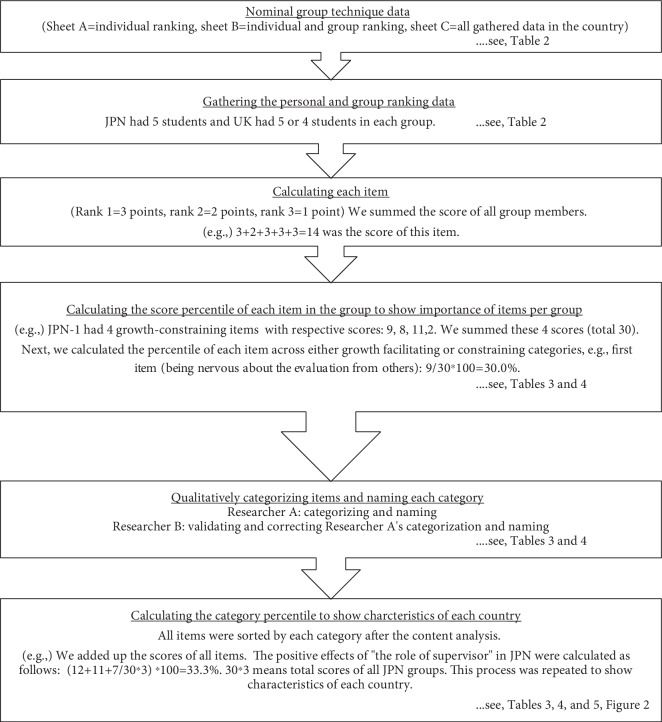
Nominal group technique process and analyses.

The NGT [9] was used to gather individual and group perspectives (Figure 1, Table 2). The NGT is a structured approach to document small-group discussions to reach overall consensus [10]. The NGT gathers information by asking individuals to respond to questions posed by a facilitator and then asking participants within each small group to discuss and prioritize the ideas or suggestions of all group members, with the latter process being repeated across all groups to obtain consensus. The process prevents the domination of the discussion by a single person, encourages all group members to participate, and results in a set of prioritized solutions or recommendations that represent the groups' preferences. The NGT is a useful method to gain group consensus because it generates a greater number of ideas than traditional group discussions and diminishes competition and pressure to conform based on status within the group [9, 11]. This format allows for individual response sheets to be incorporated into a final decision, which prevents participants from unduly influencing others' opinions. Accordingly, the NGT was employed as a method conducive to gathering individual students' perspectives and a unified method for generating the group's perspectives in ways that afforded comparison between the two countries.

We employed research collaborators (RCs) who were not directly associated with this study or assessment of potential participants as facilitators of the NGT. The RCs of the Japanese and UK sites were provided with the same guides, in Japanese and English, respectively, and were instructed to follow the rules and directions contained in the guide (Table 2). Researchers provided guidance to the RCs and answered questions from the RCs on managing the NGT groups. However, to avoid influencing the NGT, researchers did not get involved directly in the management of the NGT.

Participants were allocated to groups of four or five students within the NGT session. The NGT was conducted in four steps. The first three steps involved group discussion time in the small groups, with all groups seated around separate tables in the same space. The fourth step involved debating the top three growth facilitators and growth constraints within each group.

In the first step, each participant was first invited to identify three things that facilitated their growth and three things that constrained their growth, with no debate within the group at this point (sheet A of Table 2). We explained each growth factor to participants as follows: growth-facilitating factors were things that were believed to assist their growth during the practice placement; growth-constraining factors were things that were believed to prevent their growth during practice experiences. Each student then ranked the facilitators and constrains in order of importance (ranking 1 as most important, 3 relatively least important).

The second step was a round-robin process of feedback and discussion. Each group of participants identified the top-ranked item from each member's “facilitator” list and “constraint” list to generate a group list. Points from all the group members were then totaled for each item to produce a group item score. The items within each of the two lists were then sorted in order of hierarchy of the group's item scores. This process continued until all participants' ideas had been documented. Each recorded idea was then discussed to determine clarity and importance. The same number of ideas as there were participants in a group were recorded; however, if two ideas overlapped, participants integrated them into one idea after discussion.

In the third step, participants were asked to individually rank the selected growth-facilitating and growth-constraining items, using sheet B (see Table 2) which included the group members' items. Participants were instructed “not to automatically think of your own idea as being number one, but to consider everything from a broad perspective.” The rankings were then totaled to identify the ideas that were rated highest by each group.

The fourth step was an evaluation by the RCs of all groups' “top three items” from both lists (using sheet C, see Table 2); the results of which were presented to all participants. The whole group then reflected on the results. The ideas most highly rated by the group were then defined as the most favored group actions or ideas (Table 2).

The data gathering process of the NGT totaled forty-five minutes. The RCs collected all sheets from the participants when the NGT was completed. These sheets were then transferred from the RCs to one of the researchers. The NGT sessions in the UK and in Japan were conducted on two different days, but results were only shared to researchers after both investigations were completed. This occurred to avoid influencing the results of the other research site.

### 2.3. Analysis

Analyses were performed primarily on sheet B (both individual and group sheet), while sheet C was used only for participants' feedback. The facilitative or constraining items of self-growth between JPN-S and UK-S were collated and contrasted. For each group in each country, each item score was noted, the sum of scores within a factor was determined, and then the percentage contribution of each item (per group) as a growth-facilitating or growth-constraining factor was calculated. See Figure 1 for method of calculation.

Qualitative data were analyzed following content analysis [12]. As results of the NGT were words and short sentences, the data were not coded but instead directly categorized by a collaborator experienced in qualitative research. Throughout the categorizing process, similarities among items were used to create categories; if an item seemed to have more than one meaning, the collaborator discussed with the RCs the appropriate categorization of the item. Through discussions among all coresearchers, category names were agreed upon and refined. Finally, for each category, the percentage of points accounted for by facilitative and constraining items were calculated (refer to Figure 1).

## 3. Results

There were fifteen JPN-S participants (ages = 23.46 ± 5.30 years); three had a prior employment history, 13 had a high school diploma, one had a graduate school diploma, and one had another educational history. There were 14 UK-S participants (ages = 26.79 ± 6.58 years); all had an employment history, eight were college graduates, five had a Bachelor's degree, and two had a Master's degree (Table 1).

Each of the three JPN-S groups consisted of five participants, while the three UK-S groups consisted of five, five, and four participants, respectively. For the remainder of the paper, the JPN-S groups are referred to as JPN-1, JPN-2, and JPN-3 and the UK-S groups are referred to as UK-1, UK-2, and UK-3. Due to differences in the number of group members, a direct comparison of the raw ranking points was not performed. Instead, the ranking information was converted into a score by calculating the percentage of the score of items relative to the total score for each list (growth-facilitating list and growth-constraining list) in each group, and the items were compared between the JPN-S and the UK-S.

The 24 growth-facilitating and 27 growth-constraining items were identified as fitting five categories: self-reflection, the role of supervisor, sense of responsibility, clinical knowledge and skills, and time management. These resulting categories will be explained, including differential characteristics of the JPN-S and the UK-S.

### 3.1. Self-Reflection

The category of “self-reflection” consisted of items that were related to the capacity to reflect on one's thoughts, emotions, and actions (Tables 3–5). We analyzed that student statements such as “I was able to know what sort of person I am” and “Being nervous about the evaluation from others” (JPN-S) and “I learned from mistakes” and “Avoidance and anxiety over things” (UK-S) should be categorized under “self-reflection.” This capability for reflection is central to self-regulation, self-evaluation, and self-reflection [12, 13]. These items reflect careful thought about one's own behavior and belief. Students from both countries perceived opportunities for self-reflection as growth facilitating, despite being nervous about failure and evaluation by others, but being passive, i.e., not displaying attitudes reflective of proactive behavior or independence, was considered growth constraining.

**Figure 2:**
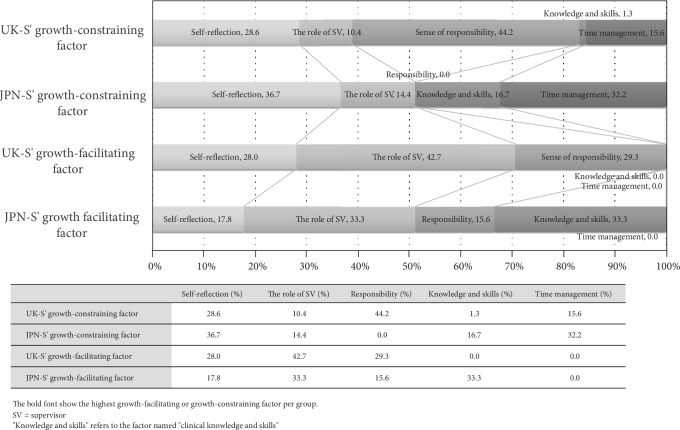
Ratio of each category per condition.

The self-reflection category accounted for the highest percentage of points of growth-constraining factors among the JPN-S (36.67%), whereas the UK-S reported nearly equal rankings for facilitating (28.6%) and constraining (28.0%) items of self-reflection (Figure 2).

### 3.2. The Role of Supervisors

“The role of supervisors” category consisted of both growth-facilitating and growth-constraining items that were related to students' relationships with their supervisors (Tables 3–5). The JPN-S and the UK-S groups reported broad similarities regarding supervisors' feedback in that it could be both growth facilitating and growth constraining. For the UK-S, constraining items included “Not being provided feedback straight away on my performance and it being provided at a later date” and facilitating items included “Talking to supervisors and other students about caseload management and patients” and “Supervision.” The UK-S thus considered receiving feedback from supervisors and practice educators as growth facilitating but felt that when the timing and quality of feedback was different from expectations, it was growth constraining. It may be implied that fear of receiving negative evaluation from supervisors prompted by the students' sharing their self-doubts or from reflections of poorer performance led to avoidance of actively seeking feedback. The category of “the role of supervisors” accounted for the highest percentage of points in the growth-facilitating factors for both countries (JPN = 33.33%, UK = 42.67%) (Figure 2).

### 3.3. Sense of Responsibility

The “sense of responsibility” category consisted of items related to responsibility for, and autonomous performance towards, the client (Tables 3–5). The JPN-S listed only one facilitating item, “I have been put in charge of a client,” while the UK-S had both constraining and facilitating items. The “sense of responsibility” category accounted for the highest proportion of constraining items in the UK-S including aspects such as “As a student, I am unable to be alone with particular patients” and “Not being able to take on enough responsibility,” while facilitating items included “Working independently.”

### 3.4. Clinical Knowledge and Skills

Students perceived clinical knowledge and skills as jointly necessary for advancing their practice placements. This category was defined as knowledge and skills for basic clinical abilities as an occupational therapist. However, clinical skills as defined in this study did not include personal attitudes, which were considered to be within the “self-reflection” category. There were clear differences in this category between the JPN-S and UK-S, with the highest scores for facilitators for the JPN-S, but no facilitators identified by the UK-S (Figure 2). Specific facilitator items included “I could make good relations with clients” and “I have thought about what is ‘occupational therapy,'” while constraint items included “Lack of confidence” and “lack of knowledge.” There were no facilitator items in this category for the UK-S, with the constraint items accounting for only 1.30% (the lowest proportion observed). The only constraint item for the UK-S was “Lacking in mental health experience.”

### 3.5. Time Management

The category “time management” consisted of items related to sleep and time for learning (Tables 3–5). For this category, students from both countries recorded only constraint items. In the JPN-S, it accounted for the second highest proportion (32.2%) of points, and in the UK-S, it accounted for 15.58% of points, for constraint items. Accordingly, the category of “time management” was only seen as growth constraining by students of both countries, although there was a qualitative difference between the two groups in the nature of the items. Specifically, for the JPN-S, the constraints were “Shortage of sleeping and learning time due to daily report and case report” and “irritation” caused by things not going as planned. We linked these reflections to students' inability to manage their time during practice placement. On the other hand, for the UK-S, constraints were predominately identified as “Not enough time to read useful resources” and “Lack of time.”

## 4. Discussion

This study set out to consider the experiences and perspectives of growth-facilitating and growth-constraining factors for learning in practice placements between JPN and UK occupational therapy. The results were analyzed from the accumulated rankings of priorities per country and topics of items compared thematically. Five themes emerged from the item content, reflecting the importance of self-reflection, role of supervisor, (sense of) responsibility, time management, and knowledge and skills. Accordingly, the following considerations are based on comparison of trends expressed in country-specific data (see Figure 2).

### 4.1. Similarities in Growth-Facilitating and Growth-Constraining Factors

There were similarities between the JPN-S and the UK-S groups in this study. Students from both countries considered opportunities for “self-reflection” as growth facilitating. However, they thought that not taking initiative, for fear of failure and/or not being “encouraged to take initiative,” as growth constraining. Previous studies have reported that student self-assessment based on self-reflection was seen as essential to the integration of educational content with life experiences to increase learning and to form a sense of competency [14, 15]. Thus, students in both countries considered that dealing with real-world experience and its associated tensions, which were only available through practice placements, provided good opportunities for learning. Fear and passivity in dealing with these situations were perceived as growth constraining. While a direct association between fear of receiving negative evaluations and avoidance of seeking feedback was not identified through the NGT, there may be connections that warrant further exploration.

### 4.2. Categories That Had Quantitative Similarities with Qualitative Differences

Facilitatory items that fell into the “role of supervisor” category accounted for the highest proportion of valued items in both countries; both groups considered feedback from the supervisor and clinical staff to be growth facilitating. For the students, regular performance feedback was considered important in order to take advantage of their placement experience with benefits of feedback identified as increased student confidence, motivation, and self-esteem as well as improving practice skills in the clinical setting [16]. Quinton et al. suggested an approach to reflective learning that recognized the need for students to engage with feedback, to reflect on it, and to feed reflections to the next assessment, thus completing the learning cycle [17]. Our findings are consistent with this approach but also suggest that responsibility for useful feedback on practice placements lie both with students and supervisors. “The feeling of participating in the feedback” reflects the transactional nature of feedback and suggests that students regarded the daily feedback as growth facilitatory.

Although opinions about supervisor feedback were similar between these two countries at a quantitative level, there were qualitative differences. For example, the UK-S placed importance on timing and quality of feedback, which may depend on the supervisor's educational ability. A previous study suggested that selection of a supervisor was often based on availability or seniority rather than demonstrated skills such as clinical expertise, effective communication, interest in students' professional growth, effective teaching skills, and/or commitment to supervision [1]. Thus, suboptimal quality and timing of feedback might be more common than intended during practice placement. Accordingly, the UK-S might experience anxiety and be confused when seeking supervisor feedback in anticipation of different opinions between supervisors. This aspect was not mentioned by the JPN-S. There is a possibility that the JPN-S prioritize supervisors' feelings in accordance with practice placements being rooted in a traditional apprenticeship model [4], a hierarchical model influenced by existing Confucianism values [18]. Therefore, the JPN-S might not report issues with the timing of and/or anxiety for feedback as they consider feedback the responsibility of their supervisors to provide in good faith.

Second, “time management” items were similar between the two countries quantitatively, but again with qualitative differences. Although all students indicated insufficient time to complete assignments during practice placements, it was notable that reasons suggested for the students' time-management problems differed between countries. The UK-S indicated that difficulties with time management pertained to the hours during which they were at the placement, while the JPN-S perceived this to be a problem that affected activities outside of practice placements, including sleep, i.e., reduced hours of sleep for study and homework such as working on assignments pertaining to their placement. As a result, the JPN-S reported stress (irritation) and expressed feelings of frustration with the challenge of persevering in these conditions. The UK-S felt that “Lack of time” for learning in practice placement was a growth-constraining factor. Why, if the content and goals of practice placements were so similar, were there differences in the experiences between the UK-S and the JPN-S regarding time management? Although the requirements for placement assignments were not different between the two universities in this study, the JPN-S may have felt a need to spend more time on their assignments to be evaluated positively. Also, under the traditional apprenticeship practice placement model, with its hierarchal structures, the JPN-S may have had less control over their goals which may impact their time management. Designing practice placements for international student needs to consider demands and expectations for time management as it relates to achieving specific placement goals. It is important that university faculty adjust the educational demands of practice placements in collaboration with supervisors, particularly report writing and the need for self-study as home-based work. Moreover, the setting of students' tasks warrants the application of pedagogical approaches such as peer learning in practice placements, which has the potential to improve “time management” [19] by students themselves. Peer teaching and peer learning can increase student's confidence in practice placement and improve learning across practical, cognitive, and communication skills for professional development [19]. Peer learning may facilitate the organization of large amounts of clinical information and knowledge and may facilitate task prioritization. For participants in this study, peer learning was introduced in practical training on campus at both universities before practice placements. However, peer-learning opportunities were not actively provided during these practice placements. Determining the ways in which peer learning could affect improvement of time management in practice placement warrants further research.

### 4.3. Differences in Growth-Facilitating and Growth-Constraining Factors

Two clear differences emerged between the JPN-S and the UK-S in this study. Firstly, differences were related to the students' sense of responsibility for the therapy they provided. Items in the “sense of responsibility” category accounted for the highest proportion of points in the growth-constraining list for the UK-S. For these students, being in charge of a client was not growth facilitating by itself, as interacting with clients without being given much responsibility was perceived as growth constraining. Here, we observed students' ambitions to become autonomous occupational therapists. In reality, students cannot hold legal responsibility for the clients as they are not yet qualified professionals. Hence, they aspire to have some level of practical and moral responsibility that helps them become occupational therapists and independently make clinical decisions. Some studies have focused on the process of building occupational therapy students' independence and responsibility through fieldwork models [20, 21]. These new forms of practice placement are aimed at improving students' moral responsibility to their clients [22] by incorporating elements of autonomous discussion and peer support, as compared with the traditional one-to-one student-supervisor interactions. Although nontraditional models of practice placements' education have been used for more than 20 years, a perception remains that these types of experiences are inferior to traditional placements [3]. However, these placements may provide students with a unique opportunity to take on responsibility more readily and thereby effectively prepare them for professional practice [23], including nontraditional practice areas [8, 24].

On the other hand, the JPN-S considered that merely being assigned a client was growth facilitating. At the same time, the JPN-S demonstrated worry and apologetic feelings towards clients (see Table 4, “I was worried that I might bother a client” in the self-reflection category), which might reflect a national tendency of the Japanese people for being self-deprecating [25]. Additionally, the fact that many clients were persons older then themselves may influence students' perceptions. In Japanese culture, the tradition of obedience towards persons older than oneself may have resulted in the JPN-S lacking self-confidence in their abilities to meet their clients' needs. This may have led to an “apologetic feeling,” with the students perceiving that they could not fulfill the role of competent health professional adequately. Differences in students' personal experiences might also have influenced their perspectives. Twelve out of 15 JPN-S did not have a prior employment history, while all UK-S had such experience. This may have contributed to the UK-S level of perceived maturity, resulting in them feeling more confident in the service provider role than the JPN-S.

Second, the significance of clinical knowledge and skills was different between the two groups. For the UK-S, lack of experience in a certain area during the practice placements was the only factor in this category that constrained their growth. It seems that the UK-S felt that “clinical knowledge and skill” before the practice placement had a low impact on their own growth compared to learn something through the practice placement. They might have felt that they had a good grounding in these skills from their university education for commencing their placement. For the JPN-S, preparatory learning before the practice placement was linked to success in placement, impacting on the experience as facilitating or constraining growth.

## 5. Limitations

Limitations to this study included the small numbers of students from the two cohorts. Therefore, comparisons made cannot fully represent all students in Japan and the UK. There was also the risk of selection bias as students who had a more positive experience on practice placement may have been more interested in participating in this study. Similarly, we did not gather any information on how the students were perceived by supervisors (e.g., positive or successful).

Nevertheless, this study offers intriguing insights into professional development through practice placement opportunities across cultures. Further study is warranted to explore how perceptions are linked to students' attitudes and actual behaviors in the practice placement setting. Results from such studies could be useful for the improvement of occupational therapy student training and progress the nurturing of students in international exchange programs.

## 6. Conclusion

This study explored similarities and differences of growth-facilitating and growth-constraining factors during practice placements, as perceived by JPN-S and UK-S. Students from both countries perceived that the opportunity to self-reflect and receive feedback from their supervisors as growth facilitating and passive attitudes towards the requirements of practice placements as growth constraining. The UK-S demonstrated a strong desire to be independent and responsible, but difficulties in time management within their placements were considered growth constraining. On the other hand, the JPN-S tended to sacrifice private time for learning and skill development. These differences may be explained by the influence of different social norms and expectations of JPN-S and UK-S. These similarities and differences should be considered when designing international learning opportunities.

## Figures and Tables

**Table 1:** Demographic data of participants.

	Students of Japan	Students of United Kingdom
Placement terms	16 weeks (8 weeks × 2 settings)	14 weeks (1 setting)
Educational form	Traditional form: learn basic occupational therapy processes and skills, with students required to assess and provide occupational therapy	
Placement level	This is a final placement for our students. This placement level demands the ability to practice under supervisors' instruction	This is a final placement for our students. This placement level demands a high level of professional integration of skills, knowledge, values, and strategies
Purpose of placement	This placement is aimed at providing students with practice from initial occupational therapy assessment through treatment to reassessment	This placement is aimed at preparing the student for subsequent practice as an occupational therapist
Gender		
Female	14	14
Male	1	0
Age years ± standard deviation (range)	23.47 ± 5.30 (21-42)	26.79 ± 6.58 (20-40)
Educational background		
High school	13	0
College (diploma)	0	8
BA/BSc	0	5
MA/MSc	0	1
PhD/doctorate	1	0
Others	1	0
Work experience		
Yes	3	14

**Table 2:** NGT methods.

NGT methods		
1^st^ step	Recording ideas: participants concisely record each idea without debate at this point	Sheet A
	Participants were divided into groups of four or five students (three groups each in Japan and the UK)	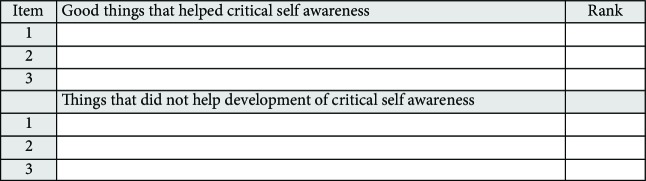
	Participants described own opinion using sheet A	
	(a) Think of three good/best things about the final placement that helped/enabled (facilitated) one's growth in critical self-awareness	
10 minutes	(b) Think of three things that participant thinks did not help/hindered (constrained) one's growth in critical self-awareness	
	(c) Rank each item on the two lists in order of importance—1st, 2nd, and 3rd	

2^nd^ step	Round-robin feedback and discussing ideas:	Sheet B
	Participants discussed in each group using sheet B	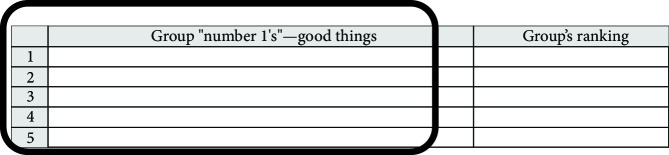
	(a) Write down the “No.1” from the “good (facilitating)” list from everyone in the group	
	(b) Write down the “No. 1” from the “hindered (constraining)” list from everyone in the group	
	Proceed until all participants' ideas have been documented	
10 minutes	Each recorded idea was then discussed to determine clarity and importance	
	All ideas of participants in a group were written down; however, if ideas overlapped, participants integrated them into one idea after discussion	

3^rd^ step	Voting on ideas: individuals vote privately to prioritize the ideas	Sheet B
	From the two lists (facilitating and constraining things), pick out the five things that participants consider most important and rank them 1-5 in order of importance using sheet B	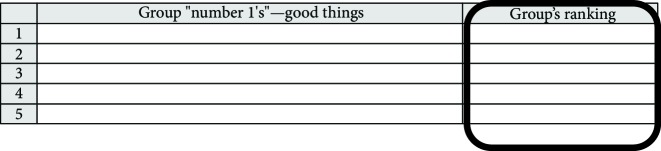
	Write down the ranking given to each item on the two lists from everyone in the group	
15 minutes	Identify the group's most important “good (facilitating)” item from placement and most important “constraining” item from placement	
	The votes were tallied to identify the ideas that were rated highest by the group as a whole	

4^th^ step	Evaluation briefs: the research collaborator (RC) collects all the cards from the participants and feedback	Sheet C
	RC wrote down all groups' “top three” (facilitating and constraining items) using sheet C	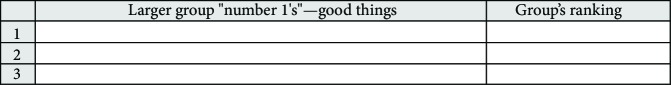
10 minutes	Whole group reconvened. RC presented the results, including any similarities and differences	
	The ideas that were the most highly rated by the group were then defined as overall the most favored facilitating and constraining items	

**Table 3:** Growth-facilitating items.

		Score	%
Self-reflection			
JPN-3	I was able to know what sort of person I am	13	43.3
JPN-3	I could express my own thoughts and feelings more easily than before	3	10.0
UK-3	I learned from mistakes	10	47.6
UK-3	Self-grading	5	23.8
UK-3	Knowing own limitations	3	14.3
UK-3	Stepping out of comfort zone	3	14.3

The role of supervisor			
JPN-1	The feedback by supervisor	12	40.0
JPN-2	The feedback by supervisor	11	36.7
JPN-2	Supervisor respected the student's autonomy	7	23.3
UK-1	Talking to supervisor and other OTS regarding caseload management and patients	13	43.3
UK-1	Feedback from practice educator and other staff	1	3.3
UK-2	Supervision	12	50.0
UK-2	Discussing my performance with practice educator directly after patient contact	5	20.8
UK-2	Talking to supervisor and other OTS regarding caseload management and patients	1	4.2

Sense of responsibility			
JPN-2	I have been put in charge of a client	12	40.0
JPN-3	I have been put in charge of a client	2	6.7
UK-1	Working independently	11	36.7
UK-1	Being trusted to “get on with it”	5	16.7
UK-2	Being in charge of a caseload	6	25.0

Clinical knowledge and skills			
JPN-1	I could make good relations with clients	9	30.0
JPN-1	I could build up relation with clients and see their smile	5	16.7
JPN-1	I could make notes about what I did not know clearly and what supervisor advised me	4	13.3
JPN-2	I have thought about what is occupational therapy	10	33.3
JPN-2	I could practice what I have learned	2	6.7

Points from all the group members were then added up for each item to produce a group item score. Percentage showed the ratio of the ranking-based score, the higher score item was regarded as an important idea in each group. Gray colored line shows each category, and following lines show labels which each Japanese and British students' group has written down. For example, JPN-1 is Japanese students group 1 and UK-1 is British students group 1.

**Table 4:** Growth-constraining items.

		Score	%
Self-reflection			
JPN-1	Being nervous about the evaluation from others	9	30.0
JPN-1	I could not be considerate of clients and other staff because of pressure from tasks and evaluation	8	26.7
JPN-2	I was passive	1	3.3
JPN-3	I was worried that I might bother a client	8	26.7
JPN-3	I was passive	7	23.3
UK-1	Finding it difficult to ask for feedback because of worry about negative comments	8	28.6
UK-3	Avoidance and anxiety over things	9	37.5
UK-3	Concerns about not doing things right, preventing opportunities	5	20.8

The role of supervisor			
JPN-2	The feedback by supervisor	7	23.3
JPN-2	Different opinions and feedback between supervisors	6	20.0
UK-1	Not being provided feedback straight away on my performance and it being provided at a later date	7	25.0
UK-2	Not being provided constructive criticism about written work completed on placement	1	4.0

Sense of responsibility			
UK-2	As a student unable to be alone with particular patient	11	44.0
UK-2	Never completely alone with a patient	7	28.0
UK-2	Not being able to work independently	6	24.0
UK-3	Not being able to take on enough responsibility	6	25.0
UK-3	Not being given opportunities	4	16.7

Clinical knowledge and skills			
JPN-1	Lack of confidence from a lack of knowledge	11	36.7
JPN-3	I could not inform client of the present state and purpose of assessment	4	13.3
UK-1	Lacking in mental health experience	1	3.6

Time management			
JPN-1	Fatigue and shortage of sleep	2	6.7
JPN-2	Shortage of sleeping and learning time due to daily report and case report writing	14	46.7
JPN-2	Unusual daily life pace	2	6.7
JPN-3	Fret/irritation	8	26.7
JPN-3	I hoped the practice placement would finish as soon as possible	3	10.0
UK-1	Not enough time to read useful resources	9	32.1
UK-1	Spending lots of time researching specialist (e.g., heart) conditions and not focusing on the OT process	3	10.7

**Table 5:** Quantitative similarities and differences between two countries.

Similarities	Self-reflection	Both students: (F) “I learned from mistakes,” “I was able to know what sort of person I am” Both students: (C) “Being nervous about failure and evaluation from others”
	The role of supervisors^∗^	JPN-S: (F) (C) “The feedback by supervisors” UK-S: (F) “Feedback from supervisors and practice educators” UK-S: (C) “The timing and quality of feedback did not meet expectations”
	Time management^∗^	JPN-S: (C) “Shortage of sleeping and learning time due to daily report and case report” UK-S: (C) “Not enough time to read useful resources”

Differences	Sense of responsibility	JPN-S: (F) “I have been put in charge of a client” UK-S: (F) “Working independently” UK-S: (C) “Not being able to take on enough responsibility”
	Clinical knowledge and skills	JPN-S: (F) “I could make good relations with clients” JPN-S: (C) “Lack of confidence from a lack of knowledge” UK-S: (C) “Lacking in mental health experience”

^∗^There were qualitative differences in this category. (F): growth facilitating, (C): growth constraining, quotations are from raw data of student opinions.

## Data Availability

The datasets during and/or analyzed during the current study are available from the corresponding author on reasonable request.

## Additional Points

*Key messages*. Japanese and UK students valued self-reflection and supervisor feedback as facilitating professional growth. Japanese and UK students differed in time management and in sense of responsibility when on placement.
